# Distinct Oral Microbiome Signatures in Cognitive Impairment: Metagenomic Insights from the Elderly MiaGB cohort

**DOI:** 10.1002/alz70856_101631

**Published:** 2025-12-25

**Authors:** Rohit Shukla, Vivek Kumar, Dhananjay Yadav, Peter Holland, Michal M Masternak, Corrie A Labyak, Mariana B. Dangiolo, Mark E. Agronin, MiaGB consortium, Hariom Yadav, Shalini Jain

**Affiliations:** ^1^ University of South Florida, Tampa, FL, USA; ^2^ Center for Microbiome Research, Microbiomes Institute, University of South Florida, Tampa, FL, USA; ^3^ Department of Neurosurgery and Brain Repair, Morsani College of Medicine, University of South Florida, Tampa, FL, USA; ^4^ FAU Schmidt College of Medicine, Boca Raton, FL, USA; ^5^ University of Central Florida, Orlando, FL, USA; ^6^ University of North Florida, Jacksonville, FL, USA; ^7^ Miami Jewish Health, Miami, FL, USA

## Abstract

**Background:**

Over recent decades, growing evidence has highlighted the pivotal role of the microbiome in Alzheimer's disease (AD) and dementia. Studies suggests the disruptions in the gut microbiome may contribute to cognitive impairment, but the association between the oral microbiome and cognitive impairment remains unclear. This study aims to characterize the oral microbiome and investigate its role in cognitive decline among elderly participants of MiaGB cohort.

**Method:**

Whole‐genome metagenomics sequencing was performed on 368 samples (Controls: 236, MCI: 107, and Dementia: 25) collected from the MiaGB (Microbiome in Aging Gut and Brain) consortium, a multi‐site, clinical research study. The data was processed and analyzed using KneadData, MetaPhlAn, and HUMAnNnn tools.

**Result:**

Taxonomic analysis revealed an increasing abundance of the genus *Porphyromonas*, and species *Neisseria subflava*, *Neisseria sicca*, and *Streptococcus australis* from controls to MCI to dementia participants. Random forest (RF) and LEfSe analysis identified significant increase in abundance of species *N. subflava*, *Veillonella parvula*, *N. sicca*, and *Neisseria flavescens* in MCI and dementia participants compared to controls. Additionally, *Lautropia mirabilis*, *Eubacterium sulci*, and *Gemella sanguinis* species were enriched in MCI compared to Controls and Dementia participants. Genera *Porphyromonas* are associated with cognitive impairment in other studies. Also, *S. australis* and *V. parvula* and *Gemella sanguinis* has been linked to neurodegenerative diseases and infective endocarditis. Distinct microbial profiles specific to each group could serve as biomarkers to identify the risk of cognitive impairment.

**Conclusion:**

This study revealed a strong link between oral microbiome alterations and cognitive impairment. Further analysis will provide a more comprehensive understanding about the role of these microbes in cognitively impaired participants. These findings offer new insights into early biomarkers for cognitive impairment and the development of potential therapeutic approaches for the prevention and intervention of Alzheimer's disease (AD).

